# MKLNID: Identifying Melanoma-related Pathogenic Genes Through Multiple Kernel Learning and Network Impulsive Dynamics

**DOI:** 10.1007/s12539-025-00755-x

**Published:** 2025-09-22

**Authors:** Linconghua Wang, Ju Xiang, Zihao Guo, Kaixin Zeng, Min Li

**Affiliations:** 1 School of Automation, Central South University, Changsha, 410083 China; 2 School of Computer Science and Technology, Changsha University of Science and Technology, Changsha, 410114 China; 3 School of Computer Science and Engineering, Central South University, Changsha, 410083 China

**Keywords:** Biological network, Melanoma, Pathogenic gene, Network propagation, Network impulsive dynamics

## Abstract

Melanoma is a highly malignant skin cancer, and identifying its pathogenic genes is crucial for understanding its pathogenesis and developing treatment strategies. Network-based approaches effectively capture the synergistic interactions among genes and their products within biological systems, yet extracting functional insights from these complex networks remains challenging. Here, we propose a novel approach that combines multiple kernel learning and network impulsive dynamics (MKLNID) to predict melanoma-related pathogenic genes. Specifically, we construct similarity kernels of diseases and genes from the original disease-gene heterogeneous network and melanoma expression profiles. These kernels are integrated via multiple kernel learning to generate enhanced similarity networks for diseases and genes, respectively. Impulsive signals are then applied to specific nodes in the enhanced heterogeneous network, and the resulting dynamical response signatures are used to infer potential pathogenic genes. Comprehensive experiments and case analyses demonstrate the effectiveness of MKLNID in identifying melanoma-related genes. By deeply integrating heterogeneous disease networks with omics data and introducing network dynamics to simulate gene responses, MKLNID offers a new strategy for identifying melanoma-related genes, with potential implications for precision diagnosis and therapy.

**Graphical Abstract:**

**Figure:**
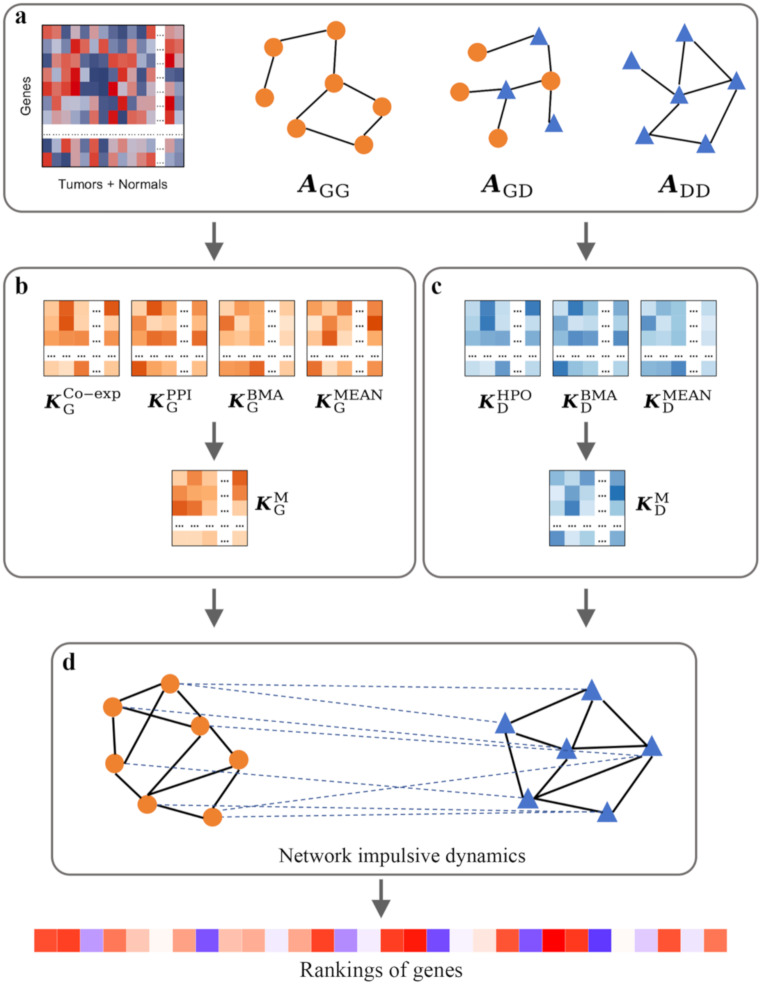

## Introduction

Melanoma is a highly aggressive tumor with over 300,000 new cases diagnosed worldwide in 2020 [1], and its incidence has been rising for decades, worryingly. Melanoma arises from pigment-producing cells, i.e., melanocytes, which are mainly located in the skin. The pigment melanin produced by melanocytes absorbs ultraviolet (UV) radiation and prevents UV-induced DNA damage in healthy skin. However, long-term UV exposure can cause somatic mutations to accumulate in melanocytes, driving oncogenic mutations. Previous studies have identified several melanoma biomarkers, such as S100 calcium-binding protein-p, MLANA and MITF, but with low specificity [2]. Whole genome and transcriptome analyses of cutaneous melanoma have revealed genomic alterations in driver genes such as BRAF, NRAS, NF1, CDKN2A, and KIT [3, 4]. In addition to UV exposure, the number of nevi, genetic susceptibility, and a family history of melanoma are important risk factors for melanoma [5]. Overall, melanoma is driven by a combination of genetic and epigenetic alterations and exhibits tumor heterogeneity, which plays an important role in phenotypic plasticity, metastasis, and drug resistance [6–8]. Despite advances in melanoma research, further efforts are required to identify additional melanoma-associated genes to improve diagnostic accuracy, prognostic assessment, and personalized treatment strategies.

Biological processes depend on the coordinated interactions among genes and their products, rather than the isolated effect of a single molecule [9, 10]. Network-based approaches provide a systematic framework for characterizing these complex molecular interactions, including protein-protein interaction (PPI) networks for physical interactions, gene regulatory networks for causal regulation, and gene co-expression networks for expression patterns [11]. Biological networks show great potential in uncovering disease mechanisms and identifying disease-associated genes. Systems biology suggests that the onset and progression of complex diseases result from structural perturbations or dynamic disruptions within local regions of molecular networks [12–14]. Advances in biotechnology have generated vast amounts of multi-omics data, providing valuable resources for elucidating the molecular mechanisms of diseases. Extracting meaningful patterns from these heterogeneous and noisy data remains a major challenge in genetics and medical research.

In recent years, network approaches have been proven effective for integrating multi-omics data and widely applied in studies of complex diseases, including melanoma [15–18]. For instance, Song et al. [19] proposed a multi-scale network analysis framework that integrates bulk multi-omics data from The Cancer Genome Atlas (TCGA) and single-cell transcriptomic data of melanoma, identifying key functional sub-networks and regulatory factors underlying melanoma progression. Ren et al. [20] constructed a heterogeneous, multi-layered molecular network by integrating various biological networks (e.g., lncRNA-mRNA, miRNA-mRNA, miRNA-lncRNA, TF-target, and PPI networks) and combined it with uveal melanoma-associated transcriptional data to identify potential susceptibility genes through network propagation. However, these studies focus on integrating multi-omics data of melanoma, while phenotypic information and its association with genetic variants remain underexplored.

Here, we propose a novel computational approach called MKLNID based on multi-kernel learning and network impulse dynamics to more effectively mine biological networks to enhance the predictive ability of identifying melanoma-related pathogenic genes. MKLNID first constructs multiple functional similarity kernels for diseases and genes based on prior biological networks and melanoma-related gene expression profiles. It then transforms kernel fusion into an optimization problem to integrate these kernels into a single kernel for diseases and genes, respectively, and build an enhanced dual-layer heterogeneous network. MKLNID initiates a network impulsive dynamics process by imposing impulsive signals at melanoma-related nodes in the heterogeneous network, and extracts the signatures of dynamical responses of gene nodes to infer potential pathogenic genes of melanoma.

The remaining work is organized as follows. In Sect. 2, we describe the biological data and the details of our method. Section 3 presents the experimental evaluation of our method, including case studies on the predicted pathogenic genes of melanoma, which demonstrate close associations between the identified candidate genes and melanoma. Finally, we give a conclusion.

## Materials and Methods

**Fig. 1:**
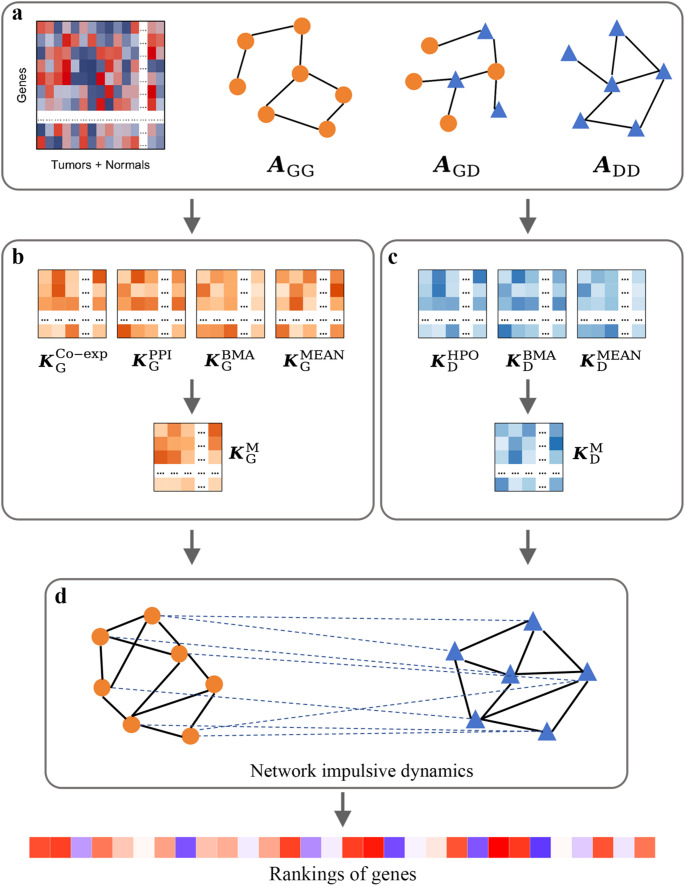
Framework of MKLNID for identifying melanoma-related pathogenic genes through multiple kernel learning and network impulsive dynamics. **a** Biological data used in this study. **b** Construction of kernels and fusion of kernels for genes based on multiple kernel learning. **c** Construction of kernels and fusion of kernels for diseases based on multiple kernel learning. **d** Network impulsive dynamics for identifying melanoma-related pathogenic genes on the enhanced dual-layer heterogeneous network

In this section, we describe the relevant biomedical data used and the details of our model’s workflow (Fig. 1).

### Biomedical Data on Pathogenic Genes and Biological Networks

To assess the predictive ability of our model and infer potential melanoma-related pathogenic genes, the datasets for high-reliability pathogenic genes and high-quality biological networks are essential. In this research, we acquire the pathogenic genes and networks from several authoritative databases or datasets.

We first collected melanoma-associated pathogenic genes from the Online Mendelian Inheritance in Man (OMIM, https://omim.org/) database, which is a well-established and exhaustive resource of human genes and genetic diseases/disorders [21]. We focused on melanoma-related phenotypes (e.g., neurocutaneous melanosis, cutaneous malignant melanoma, and melanoma-astrocytoma syndrome), and pathogenic genes (e.g., CDKN2A, NRAS, CDK4, MC1R, XRCC3, MITF, TERT, and POT1). These data served as the foundation for model training. Further, we obtained melanoma-related genes from the DisGeNet database (https://disgenet.com/), which will be used to assess model performance after training.

To characterize gene expression patterns in melanoma, we obtained gene expression profiles of normal and tumor tissues related to melanoma from TCGA and the Genotype-Tissue Expression (GTEx) databases [3, 22]. Additionally, PPI networks are the primary framework through which the biological functions of genes and their products are carried out in organisms [23, 24], reflecting the underlying associations among genes and forming a fundamental basis for exploring the molecular mechanisms of diseases. Considering the widespread data incompleteness, we utilized a high-quality and comprehensive protein interactome constructed by integrating multiple PPI sources [25], comprising only experimentally validated physical interactions.

Finally, the relationship between diseases can offer valuable information for inferring pathogenic genes. We constructed the disease similarity networks by phenotypic or symptomatic annotations of diseases, since the knowledge of diseases at phenotype/symptom levels often provides valuable insights into disease associations at gene level. Specifically, the disease similarity network was derived using the disease phenotypic annotations and the relationships between phenotypes from the authoritative Human Phenotype Ontology (HPO) database [26, 27].

### Computational Method MKLNID for Identifying Melanoma Pathogenic Genes

#### Framework of MKLNID

To more effectively identify potential melanoma-associated pathogenic genes, our computational model called MKLNID is designed to jointly extract valuable information from heterogeneous network consisting of gene-gene association (GGA) network, disease-gene association (DGA) network, and disease-disease association (DDA) network, based on multiple kernel learning and network impulsive dynamics (Fig. 1). Based on gene expression profiles of melanoma-associated normal and tumor tissues from TCGA and GTEx databases, MKLNID generates co-expression similarity kernels and constructs multiple functional similarity kernels of diseases and genes from the original heterogeneous network, respectively. Then, it integrates the similarity kernels to generate enhanced DDA and GGA networks, respectively, using multiple kernel learning. Finally, it constructs an enhanced dual-layer heterogeneous network by integrating the enhanced GGA and DDA networks with the DGA network, and infers potential melanoma-related pathogenic genes by stimulating the impulsive dynamical process excited on melanoma-related nodes, where the states of all nodes evolve over time.

#### Functional Similarity Kernels for Diseases and Genes

The biological networks for genes and diseases can provide valuable information for identification of melanoma-related pathogenic genes, but the original biological networks such as the GGA network derived from the above PPI network are incomplete and noisy, which is very unfavorable for predicting the pathogenic genes of diseases such as melanoma. Therefore, we further extract multiple similarity kernels of genes and diseases by various strategies from the original disease-gene heterogeneous network constructed by integrating the original DGA, DDA and GGA networks. For simplicity, the DGA network as a bipartite graph is denoted as $\:{\boldsymbol{A}}_{\mathrm{D}\mathrm{G}}\in\:{\boldsymbol{R}}^{{N}_{\mathrm{D}}X{N}_{\mathrm{G}}}$ ($\:{\boldsymbol{A}}_{\mathrm{G}\mathrm{D}}={\boldsymbol{A}}_{\mathrm{D}\mathrm{G}}^{\mathrm{T}}$), where the elements have values of 1 or 0 respectively for disease-gene pairs with or without known associations; the original GGA network is denoted as $\:\:{\boldsymbol{A}}_{\mathrm{G}\mathrm{G}}\in\:{\boldsymbol{R}}^{{N}_{\mathrm{G}}X{N}_{\mathrm{G}}}$, where the values of elements denote the strength of associations between genes; the original DDA network is denoted as $\:{\boldsymbol{A}}_{\mathrm{D}\mathrm{D}}\in\:{\boldsymbol{R}}^{{N}_{\mathrm{D}}X{N}_{\mathrm{D}}}$, where the values of elements denote the strength of associations between diseases.

For gene space, the first functional similarity kernel of genes is generated by

1 $$\begin{array}{l}\:{\left( {{\boldsymbol{K}}_{\rm{G}}^{{\rm{BMA}}}} \right)_{ij}} = \\\frac{{{{\sum \: }_h}{{\left( {{{\boldsymbol{A}}_{{\rm{GD}}}}} \right)}_{ih}}\mathop {{\rm{max}}}\limits_k \left\{ {{{\left( {{{\boldsymbol{A}}_{{\rm{GD}}}}} \right)}_{jk}}{{\left( {{{\boldsymbol{A}}_{{\rm{DD}}}}} \right)}_{hk}}} \right\} + {{\sum \: }_k}{{\left( {{{\boldsymbol{A}}_{{\rm{GD}}}}} \right)}_{jk}}\mathop {{\rm{max}}}\limits_h \left\{ {{{\left( {{{\boldsymbol{A}}_{{\rm{GD}}}}} \right)}_{ih}}{{\left( {{{\boldsymbol{A}}_{{\rm{DD}}}}} \right)}_{hk}}} \right\}}}{{{{\sum \: }_h}{{\left( {{{\boldsymbol{A}}_{{\rm{GD}}}}} \right)}_{ih}} + {{\sum \: }_k}{{\left( {{{\boldsymbol{A}}_{{\rm{GD}}}}} \right)}_{jk}}}}\end{array}$$

and the second functional similarity kernel of genes is generated by

2 $$\:{\left({\boldsymbol{K}}_{\mathrm{G}}^{\mathrm{M}\mathrm{E}\mathrm{A}\mathrm{N}}\right)}_{ij}=\frac{{\sum\:}_{hk}{\left({\boldsymbol{A}}_{\mathrm{G}\mathrm{D}}\right)}_{ih}{\left({\boldsymbol{A}}_{\mathrm{D}\mathrm{D}}\right)}_{hk}{\left({\boldsymbol{A}}_{\mathrm{G}\mathrm{D}}\right)}_{jk}}{{\sum\:}_{h}{\left({\boldsymbol{A}}_{\mathrm{G}\mathrm{D}}\right)}_{ih}{\sum\:}_{k}{\left({\boldsymbol{A}}_{\mathrm{G}\mathrm{D}}\right)}_{jk}}$$

where $\:{\left({\boldsymbol{K}}_{\mathrm{G}}^{\mathrm{B}\mathrm{M}\mathrm{A}}\right)}_{ij}$ and $\:{\left({\boldsymbol{K}}_{\mathrm{G}}^{\mathrm{M}\mathrm{E}\mathrm{A}\mathrm{N}}\right)}_{ij}$ denotes the elements between genes * i* and * j* in the two functional similarity kernels of genes.

For disease space, similarly, the first functional similarity kernel of diseases can be generated by

3 $$\begin{array}{l}\:{\left( {{\boldsymbol{K}}_{\rm{D}}^{{\rm{BMA}}}} \right)_{ij}} = \\\frac{{{{\sum \: }_h}{{\left( {{\boldsymbol{A}}_{{\rm{GD}}}^{\rm{T}}} \right)}_{ih}}\mathop {{\rm{max}}}\limits_k \left\{ {{{\left( {{\boldsymbol{A}}_{{\rm{GD}}}^{\rm{T}}} \right)}_{jk}}{{\left( {{{\boldsymbol{A}}_{{\rm{GG}}}}} \right)}_{hk}}} \right\} + {{\sum \: }_k}{{\left( {{\boldsymbol{A}}_{{\rm{GD}}}^{\rm{T}}} \right)}_{jk}}\mathop {{\rm{max}}}\limits_h \left\{ {{{\left( {{\boldsymbol{A}}_{{\rm{GD}}}^{\rm{T}}} \right)}_{ih}}{{\left( {{{\boldsymbol{A}}_{{\rm{GG}}}}} \right)}_{hk}}} \right\}}}{{{{\sum \: }_h}{{\left( {{\boldsymbol{A}}_{{\rm{GD}}}^{\rm{T}}} \right)}_{ih}} + {{\sum \: }_k}{{\left( {{\boldsymbol{A}}_{{\rm{GD}}}^{\rm{T}}} \right)}_{jk}}}}\end{array}$$

and the second functional similarity kernel of diseases can be generated by

4 $$\:{\left({\boldsymbol{K}}_{\mathrm{D}}^{\mathrm{M}\mathrm{E}\mathrm{A}\mathrm{N}}\right)}_{ij}=\frac{{\sum\:}_{hk}{\left({\boldsymbol{A}}_{\mathrm{G}\mathrm{D}}^{\mathrm{T}}\right)}_{ih}{\left({\mathbf{A}}_{\mathrm{G}\mathrm{G}}\right)}_{hk}{\left({\boldsymbol{A}}_{\mathrm{G}\mathrm{D}}^{\mathrm{T}}\right)}_{jk}}{{\sum\:}_{h}{\left({\boldsymbol{A}}_{\mathrm{G}\mathrm{D}}^{\mathrm{T}}\right)}_{ih}{\sum\:}_{k}{\left({\boldsymbol{A}}_{\mathrm{G}\mathrm{D}}^{\mathrm{T}}\right)}_{jk}}$$

where $\:{\left({\boldsymbol{K}}_{\mathrm{D}}^{\mathrm{B}\mathrm{M}\mathrm{A}}\right)}_{ij}$ and $\:{\left({\boldsymbol{K}}_{\mathrm{D}}^{\mathrm{M}\mathrm{E}\mathrm{A}\mathrm{N}}\right)}_{ij}$ denotes the elements between diseases * i* and * j* in the two functional similarity kernels of diseases.

Using the gene expression profiles of normal and tumor tissues related to melanoma from TCGA and GTEx, we calculate the Pearson correlation coefficient $\:{r}_{ij}$ between each pair of genes to obtain the co-expression matrices by

5 $$\:{r_{ij}} = \frac{{{{\sum \: }_h}({x_{ih}} - {{\bar x}_i})({x_{jh}} - {{\bar x}_j})}}{{{{\sum \: }_h}{{({x_{ih}} - {{\bar x}_i})}^2}{{\sum \: }_h}{{({x_{jh}} - {{\bar x}_j})}^2}}}$$

where $\:{\boldsymbol{x}}_{i}^{}$ and $\:{\boldsymbol{x}}_{j}^{}$ are the expression vectors of genes * i* and * j* that record their expression levels in multiple conditions. Then, based on the co-expression matrices, we extract the *k*-nearest neighbors of genes to generate the gene co-expression networks. These networks can reflect potential functional associations between genes, especially in melanoma, providing valuable information for identifying melanoma-related pathogenic genes.

These functional similarity kernels of diseases and genes can be used to enhance the original DDA and GGA networks by suitable approaches, and then an enhanced disease-gene heterogeneous network can be constructed by integrating the enhanced DDA and GGA networks, further improving the predictive performance of pathogenic genes.

#### Multiple Kernel Learning for Kernel Fusion

To integrate rich information in multiple similarity kernels of genes/diseases, we utilize a weighted-sum strategy to merge them into a single similarity kernel by $\:{\boldsymbol{K}}^{\mathrm{M}}={\sum\:}_{j}{\alpha\:}_{j}{\boldsymbol{{\:K}}}_{j}$, where $\:{\boldsymbol{K}}_{j}\:$ denotes the *j*-th kernel matrix for diseases or genes. The integrated similarity kernel matrix $\:{\boldsymbol{K}}^{\mathrm{M}}$ should approximate a given target similarity matrix $\:{\boldsymbol{K}}^{\mathrm{T}}$. Here, we construct the target similarity matrix using the known gene-disease network.

For diseases, we construct the target similarity matrix by

6 $$\:{\boldsymbol{K}}_{\mathrm{D}}^{\mathrm{T}}={\boldsymbol{D}}_{\mathrm{D}}^{-1}{\boldsymbol{A}}_{\mathrm{D}\mathrm{G}}{\boldsymbol{A}}_{\mathrm{D}\mathrm{G}}^{\mathrm{T}}{\boldsymbol{D}}_{\mathrm{D}}^{-1}$$

where ***A***_DG_ denotes the disease-gene association matrix; ***D***_D_=diag(***A***_DG_**1**) is a diagnal matrix for deseases, and ‘**1**’ in bold denotes a vector with all values of 1. The multiple kernel learing for gene space can be expressed as the following optimization problem:

7 $$\begin{array}{l}\:\mathop {{\rm{min}}}\limits_\alpha \:\left\| {{\boldsymbol{K}}_{\rm{D}}^{\rm{M}} - {\boldsymbol{K}}_{\rm{D}}^{\rm{T}}} \right\|_{\rm{F}}^2 + \lambda {\:_{\rm{D}}}{\left\| \boldsymbol{\alpha} \right\|^2},\:\\{\rm{s}}.{\rm{t}}.\:\:\sum_ {{j}} \alpha {_j} = 1\:{\rm{and}}\:\alpha_ {j} \ge \:0\end{array}$$

where $\:{\boldsymbol{K}}_{\mathrm{D}}^{\mathrm{M}}=\sum_{j}{\alpha}_{j}{\boldsymbol{K}}_{j}^{\mathrm{D}}$ and $\:{\lambda\:}_{\mathrm{D}}{\left|\right|\boldsymbol{\alpha}\left|\right|}^{2}$ is a regularization term used to prevent overfitting. By solving the above optimization problem, we can obtain the integrated similarity kernel matrix $\:{\boldsymbol{K}}_{\mathrm{D}}^{\mathrm{M}}$ of diseases.

For genes, similarly, we also construct the target similarity matrix $\:{\boldsymbol{K}}_{\mathrm{G}}^{\mathrm{T}}$ of genes by

8 $$\:{\boldsymbol{K}}_{\mathrm{G}}^{\mathrm{T}}={\boldsymbol{D}}_{\mathrm{G}}^{-1}{\boldsymbol{A}}_{\mathrm{D}\mathrm{G}}^{\mathrm{T}}{\boldsymbol{A}}_{\mathrm{D}\mathrm{G}}{\boldsymbol{D}}_{\mathrm{G}}^{-1}$$

where ***D***_G_=diag(***A***_DG_**1**) is a diagnal matrix for genes, and ‘**1**’ in bold denotes a vector with all values of 1. The multiple kernel learing for gene space can be expressed as the following optimization problem:

9 $$\begin{array}{l}\:\mathop {{\rm{min}}}\limits_{\alpha \:} \left\| {{\boldsymbol{K}}_{\rm{G}}^{\rm{M}} - {\boldsymbol{K}}_{\rm{G}}^{\rm{T}}} \right\|\:_{\rm{F}}^2 + \lambda {_{\rm{G}}}{\left\| \boldsymbol{\alpha} ,\right\|^2}\\\:{\rm{s}}.{\rm{t}}.\:\:\sum_ {{j}} \alpha_ {_j} = 1\:{\rm{and}}\:\alpha _{_j} \ge \:0\end{array}$$

where $\:{\boldsymbol{K}}_{\mathrm{G}}^{\mathrm{M}}=\sum_{j}{\alpha}_{j}{\boldsymbol{K}}_{j}^{\mathrm{G}}$ and $\:{\lambda}_{\mathrm{G}}{\left|\right|\boldsymbol{\alpha}\left|\right|}^{2}$ is a regularization term. By solving the above optimization problem, we can obtain the integrated similarity kernel matrix $\:{\boldsymbol{K}}_{\mathrm{G}}^{\mathrm{M}}$ of genes.

#### Network Impulsive Dynamics on Enhanced Heterogeneous Network

To extract predictive signals related to melanoma, we construct a dynamical model in a dual-layer heterogeneous network containing a gene network layer, a disease network layer and gene-disease associations as a bridge. To control the interactions between two network layers, we first define a weighted adjacent matrix for the dual-layer heterogeneous network by

10 $$\:{\stackrel{\sim}{\boldsymbol{A}}}_{\mathrm{H}}=\left(\begin{array}{cc}\left(1-\omega\:\right){\boldsymbol{K}}_{\mathrm{G}}^{\mathrm{M}}&\:{\omega\:\boldsymbol{A}}_{\mathrm{G}\mathrm{D}}\\\:\omega\:{\boldsymbol{A}}_{\mathrm{D}\mathrm{G}}&\:\left(1-\omega\:\right){\boldsymbol{K}}_{\mathrm{D}}^{\mathrm{M}}\end{array}\right)\in\:{\mathbb{R}}^{\left({N}_{\mathrm{G}}+{N}_{\mathrm{D}}\right)\times\:\left({N}_{\mathrm{G}}+{N}_{\mathrm{D}}\right)}$$

where $\:\omega\:$ is a parameter of controlling the contribution of inter-layer and intra-layer diffusions. Then, we define a Laplacian matrix of weighted dual-layer heterogeneous network:

11 $$\:\boldsymbol{L}=\mathrm{d}\mathrm{i}\mathrm{a}\mathrm{g}\left({\stackrel{\sim}{\boldsymbol{A}}}_{\mathrm{H}}\boldsymbol{1}\right)-{\stackrel{\sim}{\boldsymbol{A}}}_{\mathrm{H}}$$

where $\:{\stackrel{\sim}{\boldsymbol{A}}}_{\mathrm{H}}$ considers the contribution of inter-layer and intra-layer diffusions, and ‘**1**’ in bold denotes a vector with all values of 1. Finally, we construct the vector-based impulsive dynamical equations on the weighted dual-layer heterogeneous network by

12 $$\:{\dot{\boldsymbol{q}}}_{t}=-{\boldsymbol{q}}_{t}+\boldsymbol{L}{\boldsymbol{q}}_{t}+\boldsymbol{b}{u}_{t}$$

where $\:{\:\boldsymbol{q}}_{t}$ denotes the state of gene/disease nodes; $\:\boldsymbol{b}$ indicates which gene/disease nodes are control nodes being excited by the impulsive signal; $\:{u}_{t}=\delta\:\left(t-{t}_{\sigma\:}\right)$ is an indicator of periodic impulsive signal.

To identify melanoma-related pathogenic genes, we stimulate the impulsive dynamical process in the heterogeneous network according to the above model by exerting impulsive signals on given melanoma-related nodes, and then the response of gene nodes to impulsive excitation will be tracked as a function of time. The stronger the response of a gene node to excitation, the more likely it is to be associated with melanoma. We extract the effective impulsive dynamical signature of genes from the dynamical response of nodes to infer potential melanoma-associated pathogenic genes.

## Experimental Results

In this section, we first present the experimental settings. Then, we evaluate the performance of MKLNID model in predicting melanoma-associated pathogenic genes based on biological networks and gene expression profiles. We further investigate the effect of model parameters and functional similarity kernels. Finally, we analyze the effectiveness of predicted potential pathogenic genes for melanoma.

### Experimental Settings

To evaluate the performance of our model, we first use the known pathogenic genes in OMIM as the gold standard to serve as positive training samples, and then the melanoma-related pathogenic genes only in DisGeNet are used as positive test samples to assess the performance of the model. Multiple kinds of candidate gene sets are constructed by integrating the (positive) test genes with different control sets, including: the artificial linkage-interval (ALI) control set, the random control (RC) set, and the whole genome (WG) control set. According to the ranking list of candidate genes, we quantitatively assess the predictive performance of our model using several standard evaluation metrics. Given a list of top-*k* ranking candidate genes as the predicted positive genes, the true positive genes and false positive genes are denoted as $\:\mathrm{T}\mathrm{P}$ and $\:\mathrm{F}\mathrm{P}$, and then we calculate the standard evaluation metrics: Precision(*k*), Recall(*k*) and FPR(*k*) as a function of the length of top-*k* ranking list. Furthermore, we compute the area under the receiver operating characteristic curve (AUROC) and the area under the precision-recall curve (AUPRC), which are widely applied to evaluate the comprehensive performance of predictive models. To facilitate comparison of local performance of models, we further calculate the area under the curve of $\:\mathrm{P}\mathrm{r}\mathrm{e}\mathrm{c}\mathrm{i}\mathrm{s}\mathrm{i}\mathrm{o}\mathrm{n}\left(k\right)$ ($\:\mathrm{AUPrec}={\sum}_{1}^{{k}}\mathrm{P}\mathrm{r}\mathrm{e}\mathrm{c}\mathrm{i}\mathrm{s}\mathrm{i}\mathrm{o}\mathrm{n}\left(k\right)$) and the area under the curve of $\:\mathrm{R}\mathrm{e}\mathrm{c}\mathrm{a}\mathrm{l}\mathrm{l}\left(k\right)$ ($\:\mathrm{AURecall}={\sum}_{1}^{k}\mathrm{R}\mathrm{e}\mathrm{c}\mathrm{a}\mathrm{l}\mathrm{l}\left(k\right)$), where *k* = 100 for default.

### Performance Comparison of Predicting Melanoma-related Pathogenic Genes Based on Biological Networks

**Fig. 2:**
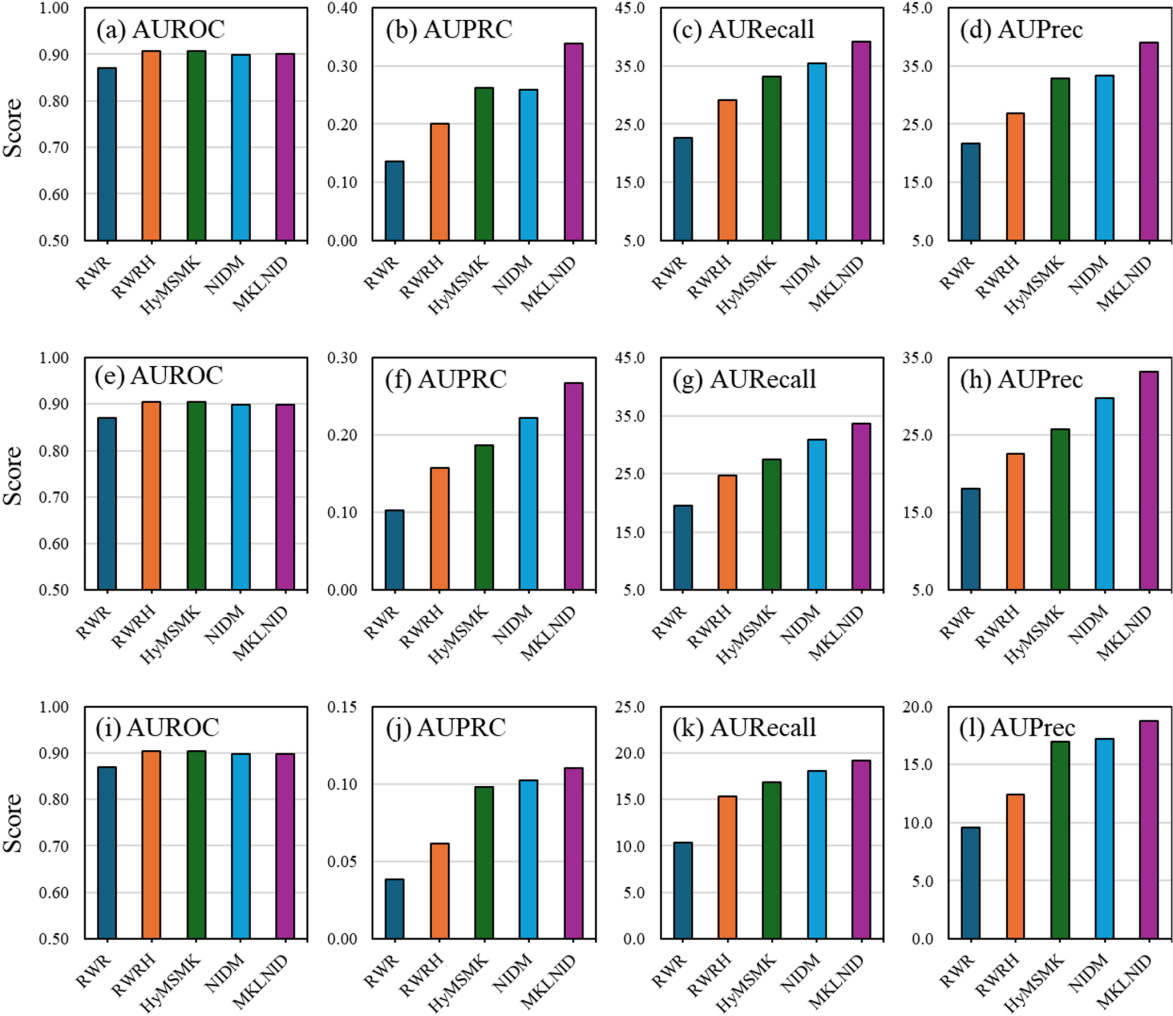
Performance comparison of our model to cutting-edge algorithms when predicting melanoma-related pathogenic genes based on only original GGA, DDA and DGA networks, under the ALI (**a**-**d**), RC (**e**-**h**) and WG (**i**-**l**) control sets

Here, we evaluate the performance of our model when predicting melanoma-related pathogenic genes only based on the original GGA, DDA and DGA networks. Figure 2 shows the performance comparison of our model to cutting-edge algorithms (RWR [28], RWRH [29], HyMSMK [30], NIDM [31]), when only the original GGA, DDA and DGA networks are used to predict melanoma-related pathogenic genes. In terms of AUPRC, AURecall and AUPrec metrics under all control sets, our model is clearly superior to the comparison algorithms, while retaining higher or comparable AUROC values. Specifically, the AUPRC values of our model are 29%, 20% and 8% higher than the best results of other algorithms, respectively, under the ALI, RC and WG control sets. The AURecall values of our model are 11%, 9% and 6% higher than those of the best comparison algorithms, respectively, under the ALI, RC and WG control sets. The AUPrec values of our model exceed those of the best comparison algorithms by 17%, 11% and 9%, respectively, under the ALI, RC and WG control sets. The results confirm that our model has superior predictive ability in predicting melanoma pathogenic genes when using only original GGA, DDA, and DGA networks as input.

### Performance Comparison of Predicting Melanoma-related Pathogenic Genes by Integrating Specific Gene Expression Profiles

Here, we further study our model’s ability to predict melanoma-related pathogenic genes when using different co-expression networks derived from normal and/or tumor tissues for melanoma. In different scenarios, we compare our model to other cutting-edge algorithms (RWRMH [31], pgWalk [32], CHN [33], NIDM [34]) using multiple networks of genes and/or diseases (Table 1). For simplicity, +Normal + Tumor denotes the co-expression network derived from normal and tumor tissues; +Normal denotes the co-expression network derived from normal tissues; +Tumor denotes the co-expression network derived from tumor tissues. The results demonstrate that our model outperforms the comparison algorithms in most cases. For example, under all three conditions above (+ Normal + Tumor, +Normal and + Tumor), for all the ALI, RC and WG control sets, our model has the highest values of AUROC compared to the comparison algorithms, meaning the best global performance for identifying melanoma-related pathogenic genes; for the ALI control set, all evaluation metrics (AUROC, AUPRC, AURecall and AUPrec) of our model are lager than the best results of the comparison algorithms. Under the ‘+Normal’ condition, for all the ALI, RC and WG control sets, the performance metrics of our model are larger than the results of comparison algorithms, except for AUPrec. The results confirm superior predictive ability of our model in predicting melanoma-related pathogenic genes when integrating co-expression networks derived from specific gene expression profiles of melanoma.

**Table 1:** Performance comparison of our model to other cutting-edge algorithms using multiple networks of genes and/or diseases. +Normal + Tumor denotes the co-expression network derived from normal and tumor tissues for melanoma; +Normal denotes the co-expression network derived from normal tissues; +Tumor denotes the co-expression network derived from tumor tissues. NIDMmx and NIDMmn denote respectively the versions of NIDM using maximum and mean values of impulsive responses

Methods			RWRMH	pgWalk	CHN	NIDMmx	NIDMmn	MKLNID
+Normal + Tumor	ALI	AUROC	0.865	0.867	0.870	0.856	0.857	**0.894**
		AUPRC	0.323	0.348	0.194	0.220	0.199	**0.361**
		AURecall	34.7	37.7	21.2	31.4	30.0	**39.4**
		AUPrec	37.7	40.0	24.2	30.8	28.5	**40.3**
	RC	AUROC	0.866	0.869	0.868	0.856	0.857	**0.894**
		AUPRC	0.261	**0.289**	0.166	0.181	0.169	0.281
		AURecall	31.7	**36.3**	17.8	28.9	26.7	34.6
		AUPrec	33.8	**36.8**	21.5	27.2	25.3	34.6
	WG	AUROC	0.867	0.870	0.870	0.856	0.857	**0.894**
		AUPRC	**0.147**	0.133	0.093	0.071	0.062	0.123
		AURecall	23.9	**25.3**	13.3	17.1	14.7	20.6
		AUPrec	**24.8**	24.1	16.9	14.8	12.5	20.8
+Normal	ALI	AUROC	0.876	0.872	0.888	0.866	0.867	**0.902**
		AUPRC	0.271	0.277	0.181	0.207	0.197	**0.347**
		AURecall	32.2	35.8	23.7	32.1	31.3	**39.5**
		AUPrec	33.6	35.5	23.3	29.8	28.3	**39.6**
	RC	AUROC	0.876	0.872	0.886	0.865	0.866	**0.899**
		AUPRC	0.209	0.198	0.135	0.190	0.163	**0.279**
		AURecall	27.7	32.6	16.9	28.8	26.8	**34.0**
		AUPrec	28.4	29.7	17.8	27.1	24.3	**34.2**
	WG	AUROC	0.876	0.873	0.886	0.865	0.866	**0.900**
		AUPRC	0.115	0.092	0.050	0.070	0.062	**0.118**
		AURecall	18.8	19.2	9.3	15.8	14.1	**19.9**
		AUPrec	**20.1**	17.8	10.9	14.2	12.0	19.9
+Tumor	ALI	AUROC	0.888	0.888	0.878	0.886	0.886	**0.895**
		AUPRC	0.282	0.323	0.185	0.248	0.229	**0.349**
		AURecall	32.4	36.3	25.7	33.5	31.9	**39.1**
		AUPrec	34.1	37.4	25.3	32.7	30.5	**39.6**
	RC	AUROC	0.888	0.890	0.876	0.886	0.886	**0.896**
		AUPRC	0.246	0.249	0.159	0.217	0.208	**0.275**
		AURecall	30.3	**34.2**	21.3	31.0	29.3	34.1
		AUPrec	31.5	33.2	22.4	30.5	28.9	**33.9**
	WG	AUROC	0.889	0.891	0.877	0.885	0.886	**0.896**
		AUPRC	**0.138**	0.113	0.086	0.097	0.086	0.117
		AURecall	20.4	**20.5**	13.6	19.8	17.0	20.1
		AUPrec	**22.5**	20.5	15.8	18.8	16.4	19.9

### Effect of Model Parameters and Functional Similarity Kernels

**Fig. 3:**
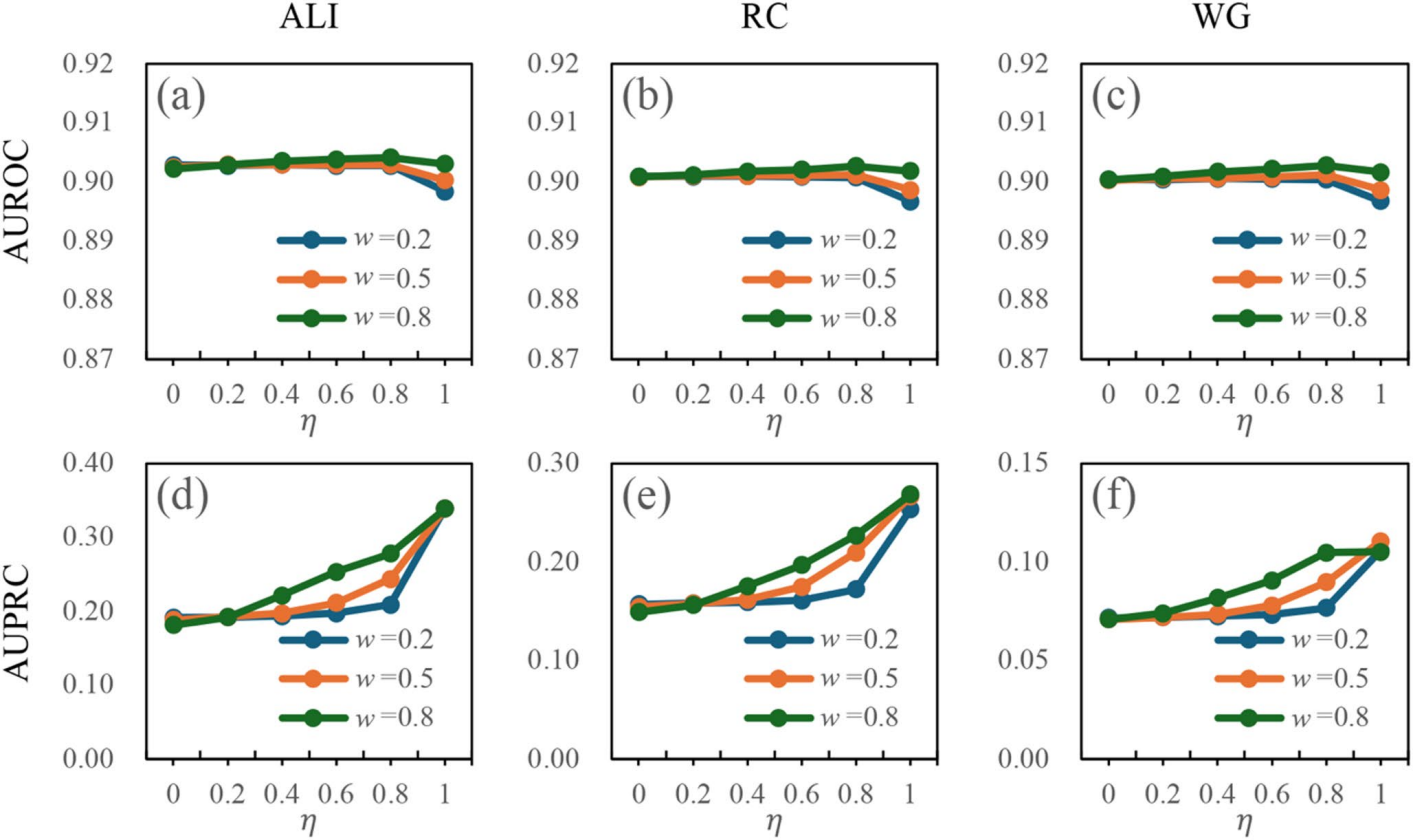
Effect of model parameters on our model’s ability to identify melanoma-related pathogenic genes under three control sets. 𝜂 is the weight of control nodes in the disease network layer; *w* is the weight of controlling diffusion between disease and gene network layers

Here, we first investigate the effect of model parameters on the predictive performance of our approach. Figure 3 shows the performance curves of model parameters in predicting melanoma-related pathogenic genes under three kinds of control sets. The parameter 𝜂 determines the weight of control nodes in the disease network layer. The parameter *w* controls the diffusion between disease and gene network layers in the dynamical process. As we see, the curves of local performance (AUPRC) significantly increase with the increase of parameter 𝜂 under all control sets, while the global performance (AUROC) has a slight decrease when parameter 𝜂 reaches a value of 1. The change of parameter *w* also has a noticeable impact on the performance of our model, especially when it is between 0.2 and 0.8. When the value of 𝜂 is close to the value of 1 or 0, the impact of *w* on AUPRC becomes very small, while the impact on AUROC is more evident. These results indicate that setting the control node(s) directly on the disease layer is the optimal choice for local performance, despite the cost of global performance degradation; parameter *w* can affect the predictive performance but the influence of 𝜂 is stronger, so we set the default value of *w* to an intermediate value of 0.5 in performance evaluation.

**Fig. 4:**
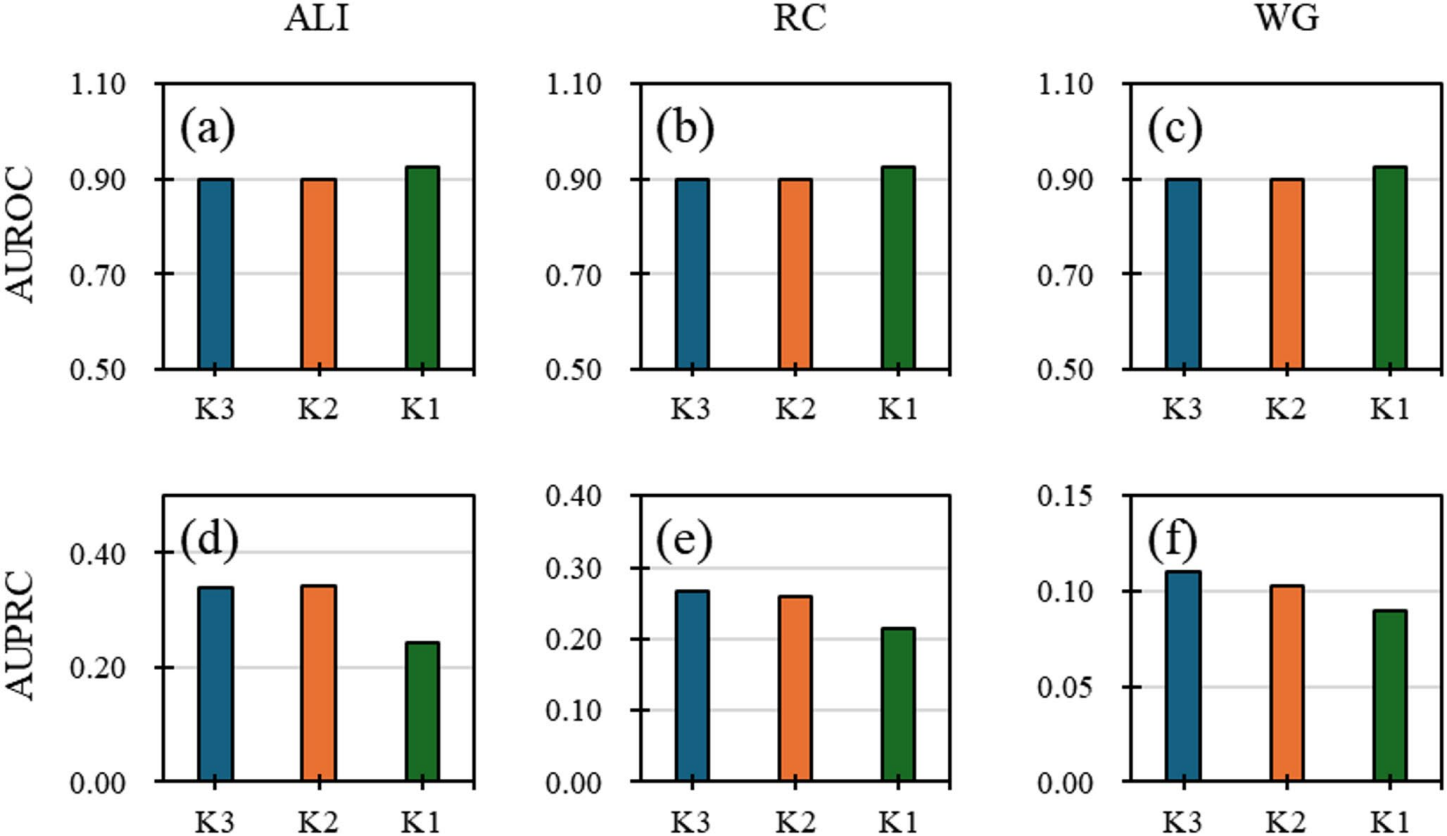
Effect of functional similarity kernels on our model’s ability to identify melanoma-related pathogenic genes when the co-expression network is unused. K3 denotes the use of PPI/DDA + BMA + MEAN kernels; K2 denotes the use of PPI/DDA + BMA kernels; K1 denotes the use of PPI/DDA

Further, we analyze the effect of two kinds of functional similarity kernels derived from the original biological networks on our model’s ability to identify melanoma-related pathogenic genes. For simplicity, the variants of our model using different functional similarity kernels are denoted as K3 (PPI/DDA + BMA + MEAN kernels), K2 (PPI/DDA + BMA kernels), and K1 (PPI/DDA). Figure 4 shows that there is a clear growing trend in AUPRC from K1 to K2 and K3, while AUROC seems to decrease slightly under three control sets. Specifically, AUPRC of K2 exceeds that of K1 by 42%, 20% and 15% under ALI, RC and WG control sets, respectively. AUPRC of K3 exceeds that of K1 by 40%, 24% and 23% under ALI, RC and WG control sets, respectively. These results indicate that the use of functional similarity kernels derived from the original biological network can lead to improvements of local performance.

**Fig. 5:**
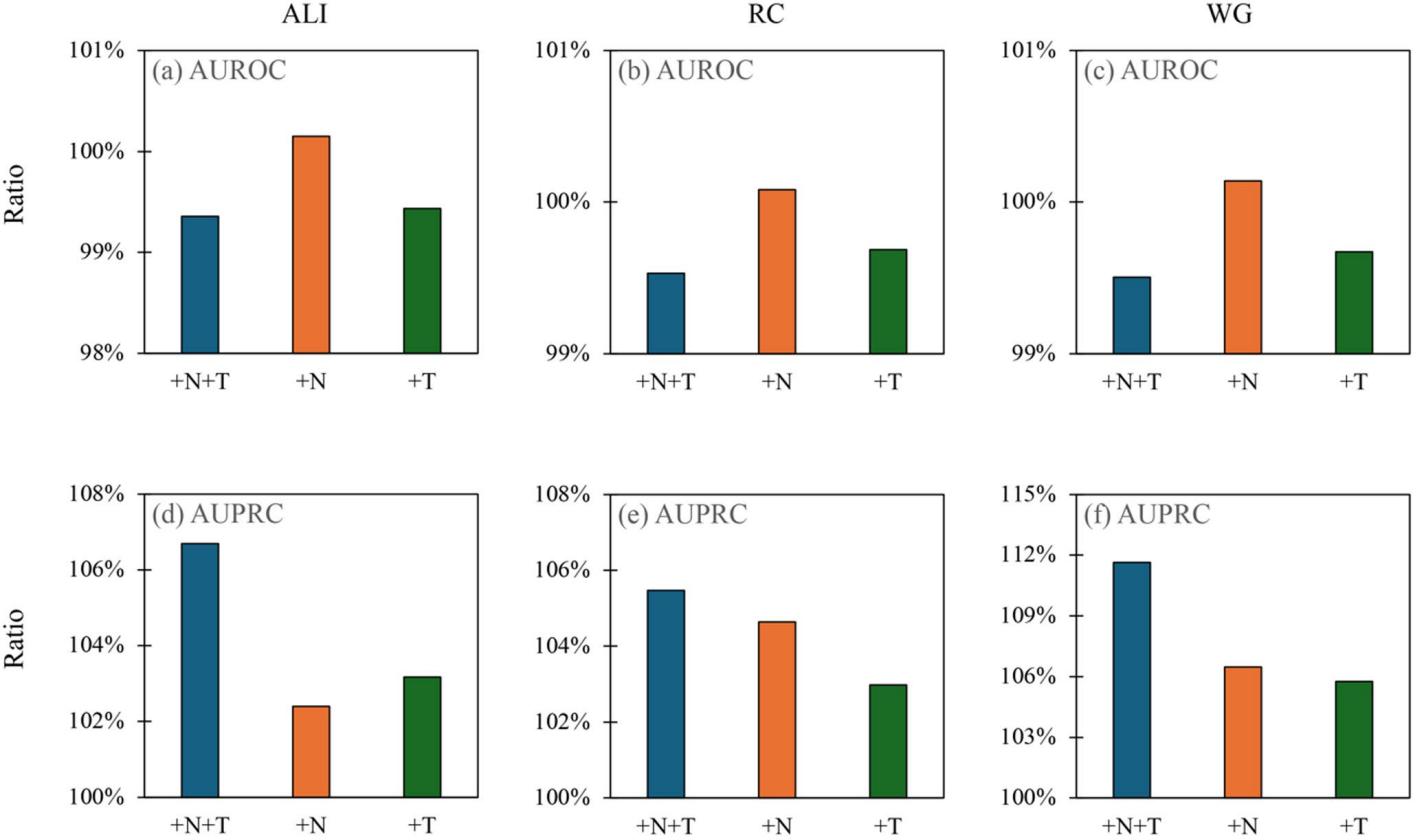
Effect of different co-expression networks derived from normal and/or tumor tissues of melanoma on the performance of our model. +N +T is to add co-expression networks derived from normal and tumor tissues for melanoma; +N is to add the co-expression network from normal tissues; +T is to add the co-expression network from tumor tissues. The *y*-axis (ratio) represents the performance of the model relative to the baseline model without co-expression networks

Finally, we analyze the effect of different co-expression networks on our model’s performance (see Fig. 5), which are constructed by the gene expression profiles from normal and/or tumor tissues of melanoma. For convenience, + N + T denotes that our model contains co-expression networks derived from normal and tumor tissues; +N denotes that our model contains the co-expression network from normal tissues; +T denotes that our model contains the co-expression network derived from tumor tissues. We tried to further use the melanoma-specific gene expression profiles to enhance the ability to identify melanoma-related pathogenic genes, while it doesn’t mean that the inclusion of any data is satisfactory. The results show that the co-expression network of normal tissues (+N) can lead to the improvement of our model’s global and local performance (AUROC and AUPRC). The co-expression network of tumor tissues (+ T) can lead to the improvement of local performance, while a slight decrease in global performance. Interestly, the co-expression network of normal + tumor tissues (+N + T) can result in the large improvement of local performance and a slight decrease of global performance. Therefore, the + N scheme is a good choice for our model if considering a compromise between local and global performance, while the +N +T scheme is a better choice if focusing on local performance (AUPRC).

### Analysis of Predicted Potential Melanoma-associated Pathogenic Genes

**Fig. 6:**
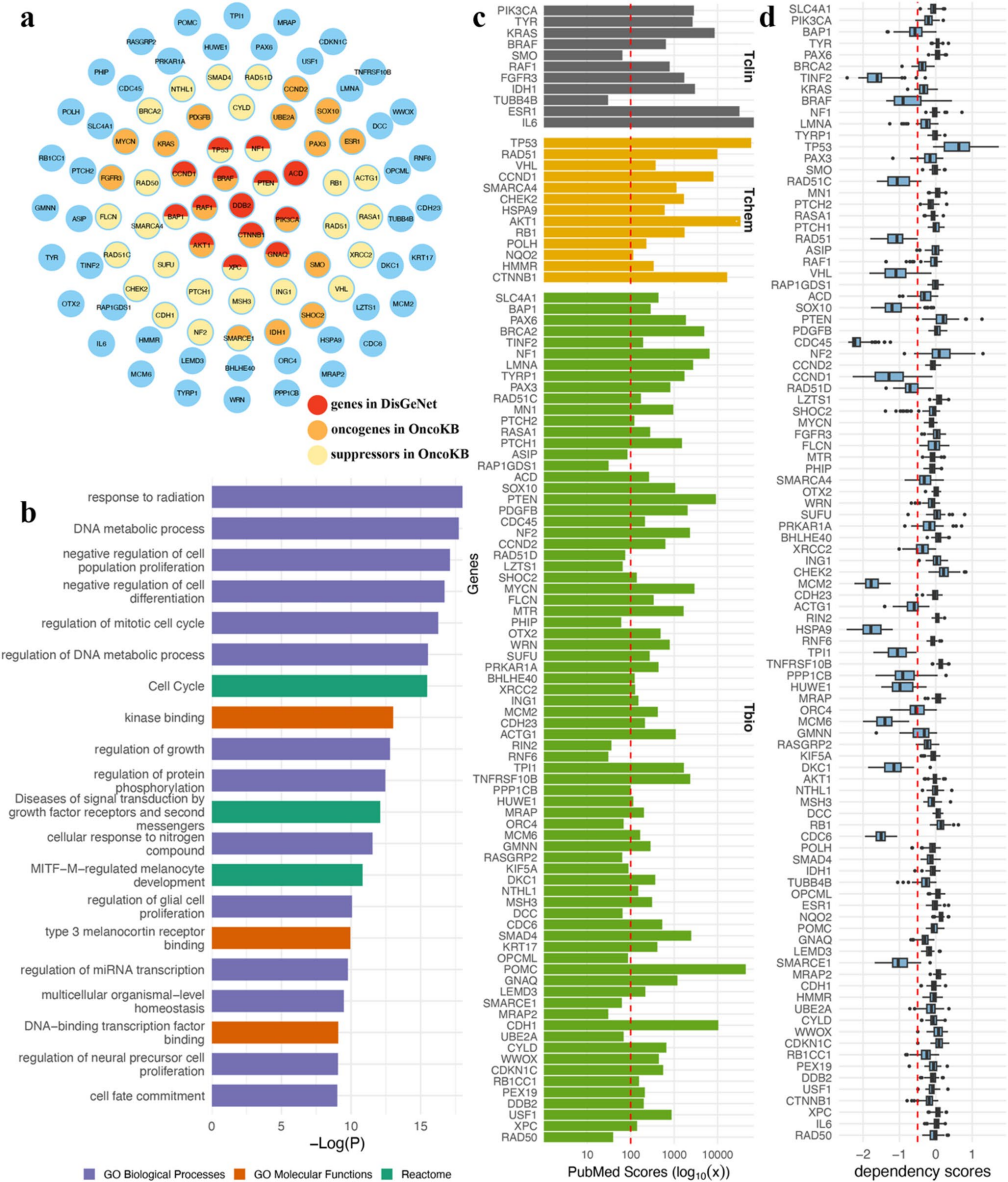
Importance analyses of melanoma-related candidate genes from our model. **a** The top 100 candidate genes according to the node prioritization. Melanoma-related pathogenic genes in DisGeNet are in red, and oncogenes and tumor suppressors in OncoKB are in orange and yellow. **b** Barplot depicting the enriched terms of the top 20 clusters across candidate genes in Gene Ontology (GO) and Reactome databases. **c** Barplot showing the summary of PubMed scores and TDLs per candidate gene (*n* = 100). The PubMed scores are log-transformed (log_10_) for visualization. **d** Boxplot showing the cancer dependency scores of candidate genes (*n* = 99) across melanoma cell lines via DepMap. Genes that not exist in DepMap are omitted. Genes with dependency scores < − 0.5 are identified as essential genes

To demonstrate the applicability of MKLNID model in inferring potential melanoma-associated pathogenic genes, we perform a case analysis based on the model’s predictions. Using known melanoma-related genes from the OMIM database as prior knowledge, MKLNID ranked all genes in the network. We then select the top 100 genes for further investigation (Fig. 6a).

Among these 100 candidate genes, 14 genes are associated with melanoma phenotypes in the DisGeNet database, while 54 genes appear in OncoKB [35], an oncology knowledge database that contains biological and clinical information about genomic alterations in cancer. We perform enrichment analysis using Metascape [36] to explore the biological roles of these genes. The results show that the candidate genes are significantly enriched in canonical cancer pathways, including cell cycle regulation, kinase binding, DNA-binding transcription factor activity, and DNA metabolism (Fig. 6b). They are also enriched in melanoma-specific pathways, such as MITF-M-regulated melanocyte development and response to radiation (Fig. 6b).

To further evaluate the potential importance of candidate genes, we compared the PubMed scores obtained from TCRD and Pharos (https://pharos.nih.gov/) [37] (Fig. 6c). PubMed scores are derived from a set of PubMed abstracts by text mining. Pharos is a public database maintained by the Illuminating the Druggable Genome Knowledge Management Centre (IDG-KMC) [38]. We find that 34% of the genes (34/100) with PubMed scores over 1000, reflecting high research interest. These include well-known cancer genes like TP53, PTEN, KRAS, RB1, and NF1. Nevertheless, 18% of genes are identified as understudied genes (PubMed score < 100) with relatively little research. We further assess the druggability of these candidate genes using target development level (TDL) categories from TCRD. Only 11% (11/100) of candidate genes are categorized as Tclin (targets of approved drugs with known mechanisms), and 13% (13/100) as Tchem (targets with bioactive small molecules meeting activity thresholds). The majority are classified as Tbio, indicating a lack of known drug or small molecule interactions. This suggests that while some genes have known therapeutic potential, many remain underexplored.

Finally, to assess the ‘essentiality’ of candidate genes for melanoma cell survival, we analyze gene dependency scores using CRISPR-Cas9 knockout data from the Cancer Dependency Map (DepMap), which is accessed through the R package DepMap (v1.6) [39]. We find that 21% of the candidate genes exhibit strong dependency (dependency score < − 0.5) in more than half of melanoma cell lines, indicating their likely importance in sustaining tumor growth (Fig. 6d), and other genes may affect cell survival through the interaction of multiple genes.

Taken together, these curated data support that candidate genes predicted by MKLNID are potentially associated with melanoma or other cancers, highlighting the model’s effectiveness and providing promising targets for further mechanistic studies and potential clinical applications.

## Discussion and Conclusion

In this study, we propose MKLNID, a novel algorithm that combines multiple kernel learning and network impulsive dynamics to effectively identify melanoma-associated pathogenic genes. MKLNID extracts multiple functional similarity kernels of diseases and genes from the gene-gene association (GGA) network, the disease-gene association (DGA) network and the disease-disease association (DDA) network to build an enhanced dual-layer heterogeneous network of diseases and genes by multiple kernel learning. To consider disease specificity, we extract co-expression similarity kernels from melanoma-related expression profiles. MKLNID further stimulates network impulsive dynamical processes by applying impulsive signals to known melanoma-associated nodes in the enhanced dual-layer heterogeneous network, and extracts the impulsive dynamical signatures of nodes’ responses to the signals as predictive scores to prioritize genes likely to be pathogenic in melanoma.

By a series of experiments, we confirmed the excellent performance of MKLNID in predicting melanoma-related pathogenic genes under diverse conditions and investigated the effect of model parameters and the contribution of different functional similarity kernels. In the case study, many of the top 100 candidate genes are significantly enriched in canonical cancer pathways. Notably, several genes, such as SOX10, TYRP1, USF1, and PAX3, are well-characterized in melanocytic differentiation and melanoma phenotypes. These results suggest the effectiveness of MKLNID in identifying biologically relevant and clinically meaningful genes.

MKLNID has some limitations. First, the construction of similarity kernels depends on public databases, which may suffer from incompleteness, outdated information, or inherent biases, despite our efforts to use high-confidence sources. Second, the current version focuses on gene expression and public networks, without incorporating other molecular layers such as DNA methylation, mutations, or copy number variations, which also play important roles in melanoma. Third, the co-expression network is constructed from bulk RNA-seq data, which does not reflect heterogeneity and microenvironment within melanoma. In future work, we can extend MKLNID as follows: incorporating omics data such as epigenetic profiles to provide a more comprehensive view, and integrating single-cell and spatial transcriptome to better characterize tumor heterogeneity and microenvironment.

In summary, MKLNID integrates heterogeneous and multi-source biological data by combining multiple kernel learning with network impulsive dynamics, enabling the discovery of richer pathogenic signals. It not only facilitates in-depth investigations of melanoma-associated pathogenic genes but also provides a generalizable and robust strategy for identifying candidate pathogenic genes across a broad range of complex diseases.

## Data Availability

This data of disease pathogenic genes and phenotypes were obtained from the OMIM database (https://omim.org/), the DisGeNet database (http://www.disgenet.org/), and the HPO project (https://hpo.jax.org/). The expression profiles of melanoma in TCGA and normal tissue in GTEx were obtained by the UCSC Xena data hub (https://xenabrowser.net/datapages/).
